# Evolution from adherent to suspension: systems biology of HEK293 cell line development

**DOI:** 10.1038/s41598-020-76137-8

**Published:** 2020-11-04

**Authors:** Magdalena Malm, Rasool Saghaleyni, Magnus Lundqvist, Marco Giudici, Veronique Chotteau, Ray Field, Paul G. Varley, Diane Hatton, Luigi Grassi, Thomas Svensson, Jens Nielsen, Johan Rockberg

**Affiliations:** 1 KTH - School of Engineering Sciences in Chemistry, Biotechnology, and Health, Dept. of Protein Science, Royal Institute of Technology, 106 91 Stockholm, Sweden; 2 Department of Biology and Biological Engineering, Chalmers University of Technology, 412 96 Gothenburg, Sweden; 3 Biopharmaceutical Development, BioPharmaceuticals R&D, AstraZeneca, Milstein Building, Granta Park, Cambridge, CB21 6GH UK; 4 GammaDelta Therapeutics Ltd, White City Place, London, W12 7FQ UK; 5 Kymab, Babraham Research Campus, Cambridge, CB22 3AT UK; 6 NBIS - Bioinformatics Systems Biology Support, Chalmers University of Technology, 412 96 Gothenburg, Sweden; 7 Novo Nordisk Foundation Center for Biosustainability, Technical University of Denmark, 2800 Kongens Lyngby, Denmark

**Keywords:** Computational biology and bioinformatics, Molecular biology, Biologics, Genomics, Metabolomics, Sequencing

## Abstract

The need for new safe and efficacious therapies has led to an increased focus on biologics produced in mammalian cells. The human cell line HEK293 has bio-synthetic potential for human-like production attributes and is currently used for manufacturing of several therapeutic proteins and viral vectors. Despite the increased popularity of this strain we still have limited knowledge on the genetic composition of its derivatives. Here we present a genomic, transcriptomic and metabolic gene analysis of six of the most widely used HEK293 cell lines. Changes in gene copy and expression between industrial progeny cell lines and the original HEK293 were associated with cellular component organization, cell motility and cell adhesion. Changes in gene expression between adherent and suspension derivatives highlighted switching in cholesterol biosynthesis and expression of five key genes (RARG, ID1, ZIC1, LOX and DHRS3), a pattern validated in 63 human adherent or suspension cell lines of other origin.

## Introduction

The production of protein therapeutics is a fast-growing field as it allows for the generation of sophisticated molecules with high specificity and activity in humans^1–4^. Even though the Chinese hamster ovary (CHO) cell line is a successfully used mammalian platform for the production of advanced recombinant proteins with the need for proper protein folding and post translational modifications, there is an increasing demand for improved and more efficient bioproduction platforms. With an increasing number of difficult-to-express proteins entering clinical development, including bispecific antibodies and antibody–drug conjugates, alternative or engineered expression hosts are being explored. Extensive omics profiling of CHO cells has been carried out during recent years^5–12^, which has paved the way for cell line engineering efforts aiming to improve bioproduction efficiency and product quality^13–15^. Moreover, human production cell lines, such as HEK293, have served as convenient expression hosts for proteins with specific requirement for human post-translational modifications^16,17^.

The human cell line HEK293 is the most commonly utilized human cell line for expression of recombinant proteins for a multitude of research applications. This cell line originate from the kidney of an aborted human female embryo and was originally immortalized in 1973 by the integration of a 4 kbp adenoviral 5 (Ad5) genome fragment including the E1A and E1B genes, at chromosome 19^18,19^. The expression of E1A and E1B enable continuous culturing of HEK293 cells by inhibiting apoptosis and interfering with transcription and cell cycle control pathways^20^. In addition, E1A and E1B are essential helper factors for adeno associated virus (AAV) production, which makes HEK293 cells attractive production hosts for recombinant AAV particles^21^. HEK293 cell lines have been reported to have a pseudotriploid genome with the adenoviral DNA inserted on chromosome 19^19,22,23^. The organization of the HEK293 genome is continuously evolving through the events of chromosomal translocations and copy number alterations, suggesting that long-term cultivation and subcloning of cells result in karyotypic drift^22,24^. Such abnormalities and genomic instability is, however, characteristic for immortalized cells and have also been reported for CHO cells^25–28^.

Several HEK293 cell lineages have been established from the parental HEK293 lineage with the objective to improve recombinant protein production and are used for the production of therapeutic proteins^16,17^. Two examples are 293T^29^ and 293E^30,31^ cell lines, constitutively expressing the temperature sensitive allele of the large T antigen of Simian virus 40^29^, or the Epstein-Barr virus nuclear antigen EBNA1, respectively^30,31^. In addition, several HEK293 cell lines have been adapted to high-density suspension growth in serum-free medium^32–34^, enabling large-scale cultivation and bioproduction in bioreactors^24^. Two industrially relevant suspension cell lines are 293-F and 293-H (Gibco, Thermo Fisher Scientific), which both enable fast growth and high transfectivity in serum-free medium. In addition, the 293-H cell line, which was originally derived from a more adherent HEK293 cell clone, shows strong adherence during plaque assays. Despite extensive usage of CHO and HEK in both suspension and adherent mode and several empirical protocols for adaptation in either direction, molecular knowledge of the key genes involved in the transition between the two growth states are limited. While adherent cells have traditionally been widely used for the production of viruses, e.g. AAV and lenti virus for clinical research, suspension growth is the platform of choice for bioproduction of therapeutic proteins. Whereas certain experimental steps are more efficient in adherent mode, e.g. chemical transfection and viral infection, the ability to increase the volumetric cell density by growth in suspension without cell clump formation, which results in oxygen limitations, is a key step from a manufacturing perspective.

Even though different HEK293 strains have all been derived from the same original cell line, significant genomic and transcriptomic changes between parental and progenitor cell lines can be expected due to the genomic instability of HEK293 as discussed above. Here, we present a genomic and transcriptomic analysis of the HEK293 parental cell line along with five widely used HEK293 derivatives. An overall analysis of the differences in genomic landscape and transcriptomic profiles was performed in order to provide novel molecular insights into the differences between cell lines that have occurred during the process of clonal isolation and expansion. Furthermore, we focus on transcriptomic differences between adherent and suspension HEK293 cells and the impact of the differentially expressed genes on metabolic pathways and the phenotype of the cells from a bioprocess perspective.

## Results

### Genomic and transcriptomic profiling indicate clonal divergence between parental HEK293 and its progeny

**Figure 1:**
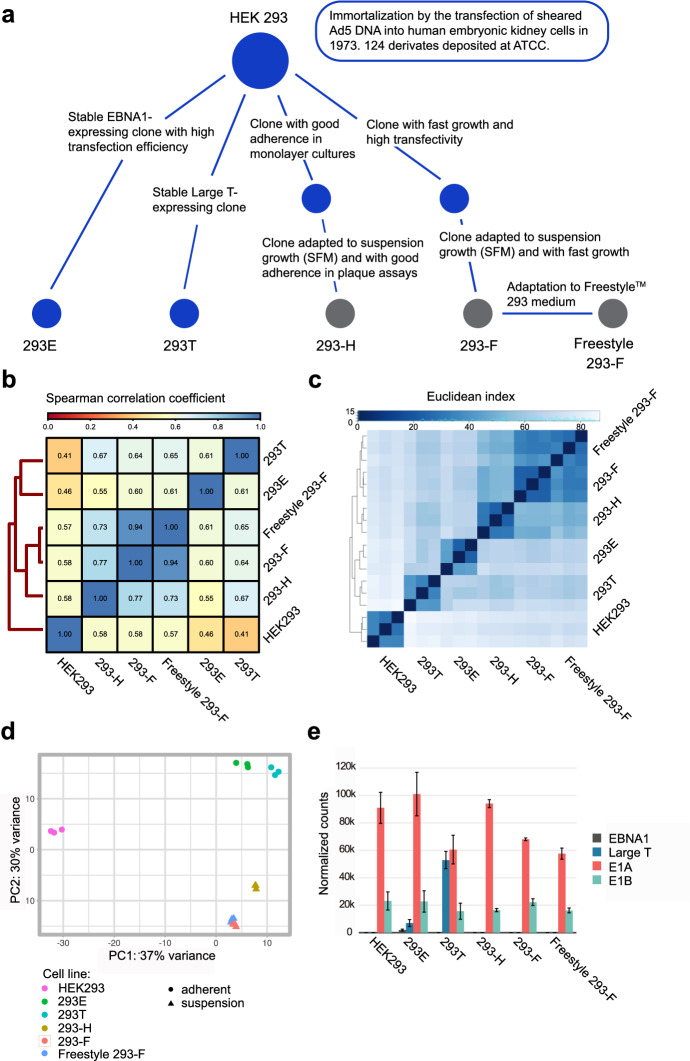
Comparisons of genomic and transcriptomic profiles of HEK293 cells showed taxonomic divergence between parental HEK293 and progeny cell lines. (**a**) A schematic overview of the lineage relationship of the six HEK293 cell lines used in this study. Blue dots represent adherent cells whereas grey dots represent suspension cell lines. (**b**) Genomic comparison between HEK293 cell lines based on Spearman correlation coefficients of read counts. Darker blue color indicates higher correlation. (**c**) Sample-to-sample comparison between transcriptomes illustrated by a heatmap and hierarchical clustering of taxonomical divergence between samples. Darker blue color indicates shorter Euclidean distance between samples and more similarity. (**d**) PCA plot showing the separation in expression pattern between samples. (**e**) RNA expression levels (in DESeq2 median of ratios) and standard deviations (n = 3) of stably integrated viral genes (EBNA-1, Large T, E1A and E1B) in HEK293 cell lineages determined by RNA sequencing.

In this study, six industrially relevant HEK293 cell lines (Fig. 1a) were subjected to omics profiling. This set of cell lines includes the parental HEK293 as well as five additional cell lines that have all been clonally derived from parental HEK293 cells. The cell lines can be divided into either adherent (HEK293, 293E and 293T) or suspension (293-H, 293-F and Freestyle 293-F) cells. The genomes and the transcriptomes of these six cell lines were sequenced using Illumina HiSeq. Supplementary Table S1 provides full results of transcript levels (TPM) for all cell lines. Comparisons of the genomes and transcription profiles between the cell lines show overall similar results (Fig. 1b,c). Hierarchical clustering divided the progeny cell lines into two different taxonomic groups, of either adherent (293T, 293E) or suspension cell lines (293-H, 293-F and Freestyle 293-F), diverged from the parental HEK293. Interestingly, the original HEK293 cell line was the most distant from all other cell lines. As expected, the two 293-F lineages (293-F and Freestyle 293-F) showed very similar profiles. The same pattern of gene expression clustering was visualized by principal component analysis (Fig. 1d), where the suspension cell-lines grouped together in the plot, with a very close clustering of 293-F and Freestyle 293-F cells. On the other hand, the adherent cell-lines 293E and 293T showed larger variations in gene expression patterns between cell lines. The parental cell line HEK293 showed a notable difference in transcriptome profile compared to all the other cell lines along the first principal component (PC1). These results indicate a genomic divergence of the clonal lineages compared to the parental HEK293 and suggest the presence of similar transcriptomic traits between HEK293 progeny cell lines individually selected for during the isolation of each clone. Hierarchical clustering of the cell lines based on SNVs gave a slightly different trend compared to the transcriptomic comparison. A different pattern of overall clustering was observed, with the original HEK293 and 293E cell lines separated from the rest on a separate branch and the three suspension cell lines grouped on a second branch together with 293T. However, 293-F and Freestyle 293-F were, as expected, the most similar cell lines also in this comparison (Supplementary Fig. S1). The overall number of genomic variations was similar between the cell lines and variations located to similar genomic regions (Supplementary Fig. S1). Moreover, the ratio between missense and synonomous SNVs in all cell lines ranged between 0.866 and 0.879, where the original HEK293 strain had the lowest ratio (Supplementary Fig. S1). Pairwise comparisons of SNVs and indels between HEK293 and each progeny cell line showed that the highest number of high (variants expected to have a disruptive impact on the protein, for example protein truncation or loss of start/stop codon) and moderate (non-disruptive variant that might change protein effectiveness, for example a missense variant) SNVs and indels were seen for the two adherent cell lines 293E and 293T (Supplementary Fig. S1). Genes with high impact SNVs in progeny cell lines compared to the parental HEK293 can be found in Supplementary Table S2. Amongst these, five genes had acquired high impact SNVs (PPP2R4, C9orf43, CTB-47B11.3, CYFIP2 and SGCD) in all progeny cell lines compared to the parental strain.

The HEK293 cell line was originally immortalized by the random integration of viral genomic DNA of adenovirus 5^18^, which includes the E1A and E1B genes. In this study, overall high mRNA levels of E1A and E1B were observed in all HEK293 cell lines (Supplementary Fig. S2). A comparison of mRNA levels of the viral element E1A showed significantly (p < 0.05) higher expression in HEK293 compared to both 293T and Freestyle 293-F. In addition, both 293E and 293-H had significantly higher expression than 293T, 293-F and Freestyle 293-F. Further, 293-F had significantly higher expression than Freestyle 293-F. (Supplementary Fig. S1) The analysis of the viral element E1B showed that 293-F had significantly higher expression (p < 0.05) than 293-H and Freestyle 293-F (Supplementary Fig. S1). As expected, the gene expression of Large T and EBNA-1 was detected in 293T and 293E, respectively (Fig. 1e). Interestingly, expression of the Large T antigen was also observed in 293E, which is not reported by the supplier (ATCC). The presence of a truncated version of Large T in the 293E genome was confirmed by de novo assembly of all reads not mapping to the human reference genome (Supplementary Fig. S2). Tracing the origin of the 293E cell line^31^, the Large T expression of 293E may be derived from the pRSVneo plasmid that was used to co-transfect HEK293 cells along with the pCMV-EBNA plasmid for the generation of the stable EBNA-1 expressing clone (293c18) by geneticin (G418) selection. The pRSVneo plasmid contains a truncated version of the Large T gene (according to the AddGene vector Database), which aligns perfectly with the truncated Large T sequence found in the 293E genome (Supplementary Fig. S2).

### Progeny cell lines displayed common patterns of copy number gain/loss at several genomic loci compared to parental HEK293

**Figure 2:**
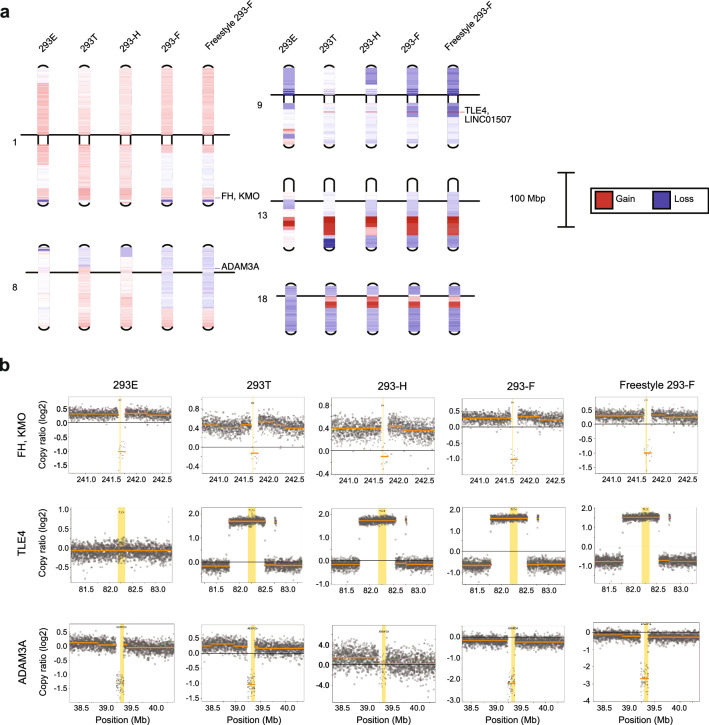
Copy number variation analysis of HEK293 progeny cells compared to the parental HEK293 revealed conserved patterns of copy number gain and loss. (**a**) Genomic copy number gain (red) or loss (blue) of chromosomes 1, 8, 9, 13 and 18 of progeny HEK293 cell lines compared to parental HEK293 cells. The black line indicates the centromere position of each chromosome. (**b**) Genomic copy number gain or loss (log2 fold change) compared to HEK293 for each cell line of the FH, KMO, TLE4 and ADAM3A genes.

In order to evaluate the genomic variation between HEK293 and its derivatives further, overall genomic copy number variation of all progeny cell lines compared to the parental HEK293 was performed. A comparison of gained and lost regions on all chromosomes between all cell lines can be found in Supplementary Fig. S3 and Table S3. Interestingly, a conserved pattern of copy number gain or loss of large regions has occurred on several chromosomes of all HEK293 progeny cells compared to HEK293, whereas other changes are more local or cell line specific. For instance, on chromosome 13, a region of > 15 Mb has been amplified in all cell lines compared to the parental HEK293 strain (Fig. 2a). All elements with copy number gain of > 1 log2 fold-change common to all progenitor cells are located in this region (Supplementary Table S3). Amongst these, four out of seven protein-coding genes (BORA, MZT1, PIBF1 and KLHL1) belong to the cytoskeleton gene set (GO: 0005856). On chromosome 18, there is a conserved pattern of copy number loss of most of the chromosome sequence for all progeny cell lines compared to the parental HEK293 strain, with the exception of a high degree of copy number gain (> 0.8 log2 fold-change) of a region close to the centromere for all cell lines except 293E (Fig. 2a). Within the region of conserved gain are several genes encoding cell adhesion molecules within the desmocollin (DSC) and desmoglein (DSG) subfamilies, belonging to the cell–cell adhesion gene set (GO: 0098609). When analyzing more local copy number variations between progeny cell lines and the parental strain, some interesting loss or gain of full or partial elements compared to the parental HEK293 were identified. For instance, copy number loss was observed for the fumarate hydratase (FH) gene, which has previously been reported to have lost several gene copies in HEK293 and hence been hypothesized to play a role in the phenotypic transformation of HEK293^22^. Interestingly, the fumarate hydratase gene along with the neighboring kynurenine 3-monooxygenase (KMO) gene, had a log2-fold copy ratio of < -1 in 293E, 293-F and Freestyle 293-F cell lines compared to the parental HEK293 (Fig. 2b and Supplementary Fig. S4), suggesting that these cells have half the number of copies compared to the parental cell line. Moreover, the 293T and 293-H cell lines have a gain of the genomic loci surrounding the FH gene, while maintaining the copy number of the FH gene compared to HEK293. Interestingly, the resulting FH expression levels of the cell lines only partly reflected the gene copy number changes (Fig. 2b and Supplementary Fig. S4). Even though the gene copy number of the parental HEK293 strain is the same as for 293T and 293-H lineages, the FH mRNA levels of HEK293 was as low as the expression levels of the lineages with only half the number of FH gene copies. Moreover, the expression levels of KMO was comparably low in all cell lines but did not correlate with gene copy number. Besides the changes in gene copy number of the FH locus, a locus around the transducin-like enhancer protein 4 (TLE4) gene, encoding a transcriptional co-repressor of Wnt signaling pathway members was found to have a log2-fold copy number gain of > 1.5 in all progeny cell lines except for 293E (Fig. 2b). This gain in the TLE4 locus was accordingly reflected in the transcription level of the gene with a higher level of expression in 293T, 293-H, 293-F and Freestyle 293-F compared to 293E and HEK293 (Supplementary Fig. S4). In addition, a major loss of copy number of the ADAM3A pseudogene was observed for all cell lines except 293-H with a maintained low or no expression of the pseudogene observed in the cell lines (Fig. 2a,b and Supplementary Fig. S4).

Due to the observed pattern of common genomic changes to progeny cell lines compared to the parental HEK293, an evaluation of common SNPs amongst all progeny cell lines but not HEK293 was performed. GO enrichment analysis of common genes with high or moderate impact SNPs different in all progeny cell lines compared to the original HEK293 (Supplementary Table S4), showed significant (adjusted p-value < 0.05) enrichment of homophilic cell adhesion via plasma membrane adhesion molecules (GO:0007156; adjusted p-value 0.025; fold enrichment 10.26; data not shown) and cell–cell adhesion via plasma-membrane adhesion molecules (GO:0098742; adjusted p-value 0.032; fold enrichment 7.53; data not shown). All genes with moderate or high impact SNPs in progeny cell lines compared to HEK293 found amongst both these GO-terms were protocadherins (PCDH12, PCDHB10, PCDHB13, PCDHB15, PCDHB16 PCDHGA2, PCDHGA3 and PCDHGB2). In addition, the Teneurin-2 gene (TENM2) (within GO:0098742) had an altered SNP allele in all progeny cell lines compared to HEK293. These SNPs all result in missense mutations with unknown biological impact on the gene products. However, the enrichment of common SNPs within this group of genes in all HEK293 progeny cell lines may suggest an impact on the protein function and a selective advantage of such phenotypic changes during continuous cell line cultivation.

### Consensus differential expression analysis suggested a role of integral membrane proteins in HEK293 progeny cell line development

**Figure 3:**
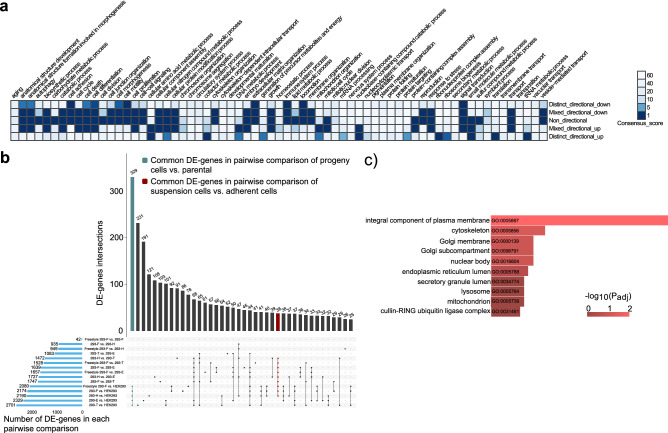
Differential expression analysis emphasized processes and genes with common changes in all progeny cell lines compared to the parental HEK293. (**a**) Consensus heatmap of GO biological processes with a different expression pattern between progeny cell lines compared to the parental HEK293. Low consensus scores, represented by a dark blue color, indicate more significant differences. (**b**) Common differentially expressed (DE) genes in pairwise comparisons of all HEK293 cells. Blue bars show number of DE genes in each pairwise comparison. Green bar shows 329 common DE genes in pairwise comparisons of progeny with HEK293 parental cells. Red bar shows common 38 DE genes in the comparison of suspension cells against adherent cells. (**c**) Top ten significant GO cellular components of the 329 common DE genes in pairwise comparisons between progeny cells and HEK293.

Based on the overall genomic and transcriptomic profiles of the different HEK293 cell lines, the parental HEK293 strain stood out as different compared to all other cell lines. In order to evaluate common changes between all progeny cell lines and the parental HEK293, differential expression analysis was performed. Results showed a significant consensus of down-regulation of genes involved in extracellular matrix organization, locomotion and cell adhesion in progeny cells compared to the parental HEK293 strain (Fig. 3a). Moreover, amino acid metabolism and metabolic process of small molecules were found up-regulated in all progeny cell-lines. Along with changes in extracellular matrix genes, there is also a consensus amongst progeny cell lines compared to HEK293 of differential expression of genes involved in other types of cellular component organization such as cell morphogenesis, cytoskeleton-, membrane- and cell junction organization. A comparison between gene expression fold changes and copy number variation of the differentially expressed genes (log2-fold change >  ± 1) for each progeny cell line compared to HEK293 showed a trend of gained gene copies amongst the majority of genes with up-regulated mRNA levels (Supplementary Fig. S4). However, there was not a clear trend of loss in gene copy number amongst transcriptionally down-regulated genes for any cell line.

For further evaluation of the transcriptomic similarities and changes between HEK293 cell lines, pairwise differential expression comparisons between all cell lines were performed. As expected, the parental cell line had the highest number of differentially expressed genes when compared to all other cell lines (Fig. 3b, Supplementary Fig. S5 and Supplementary Table S5). In addition, when looking at differentially expressed genes unique to certain comparisons, the largest group of genes were found common to all pairwise comparisons between HEK293 and each of the progeny cell lines (green bar in Fig. 3b), again emphasizing a relatively high degree of common transcriptomic changes amongst progeny cell lines differentiated from the parental HEK293. As the progeny cell lines had an enrichment of differentially expressed genes associated with cellular component organization compared to HEK293, we sought to evaluate to what cellular compartments the 329 genes common to all pairwise comparisons between HEK293 and progeny cell lines localize. In line with the overall differential expression evaluation (Fig. 3a), which emphasized changes in for instance cell adhesion and extracellular matrix organization, there was a significant (padj < 0.05) enrichment of genes relating to the integral compartment of plasma membrane (GO:0005887) amongst the common differentially expressed (DE) genes unique to the comparisons between HEK293 and all progeny cell lines (Fig. 3c). Moreover, gene set analysis of these 329 genes showed, although non-significant in this limited set of genes, alterations in processes related to cell surface, cell adhesion and epithelial to mesenchymal transition (Supplementary Fig. S6).

### Differential expression between suspension and adherent HEK293 cell lines identified key changes related to cholesterol metabolism

**Figure 4:**
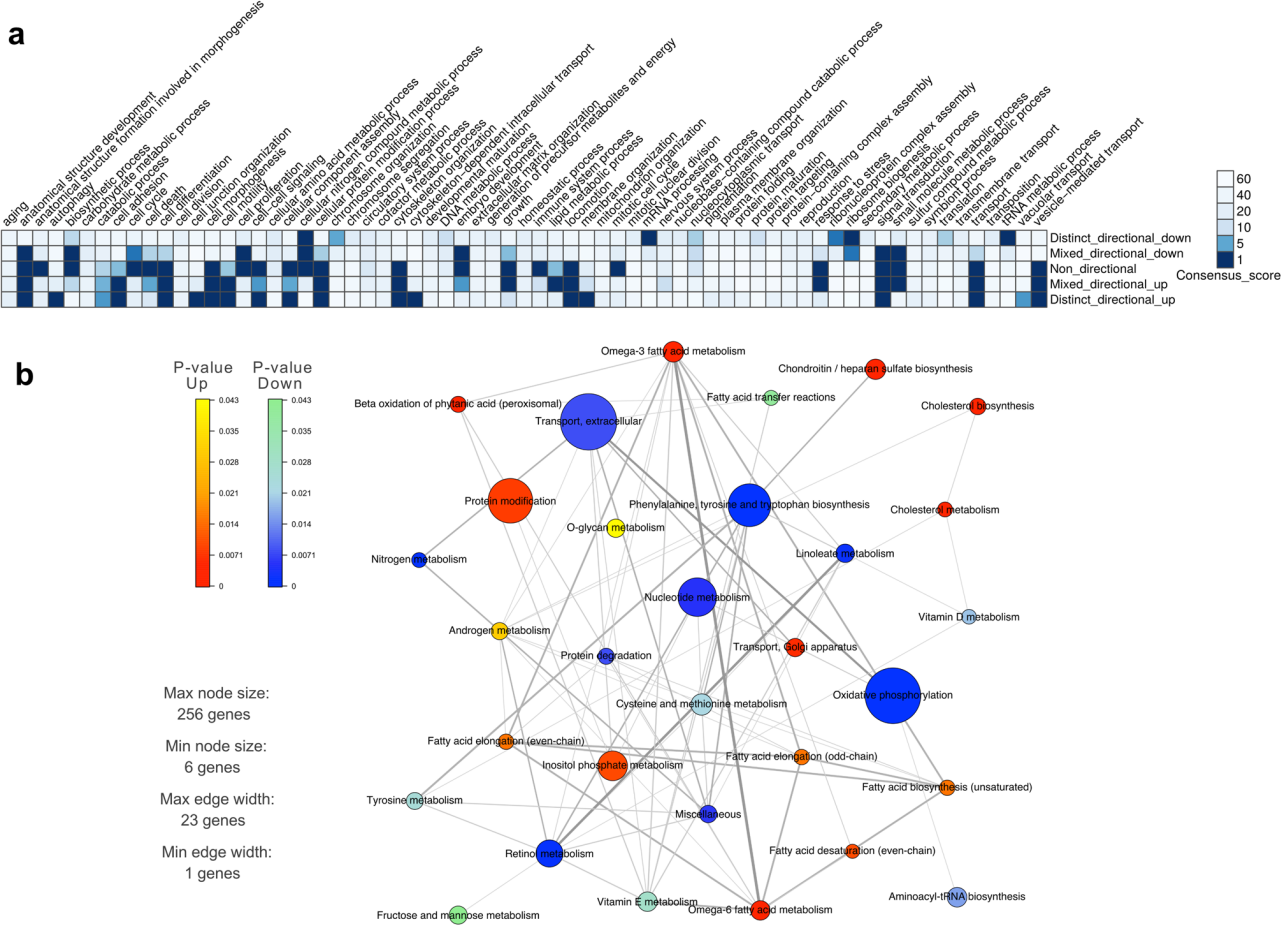
Gene set analysis identified biological process and metabolic changes between suspension and adherent HEK293 progeny cell lineages. (**a**) Heatmap of gene set analysis using GO biological processes and Piano consensus scores showed a different expression pattern between suspension cells (293-H and 293-F) and adherent cells (293E and 293T). Low consensus scores, represented by a dark blue color, indicate more significant differences. (**b**) Metabolic genes set analysis for comparing metabolic differences between suspension and adherent HEK293 progeny cell lines. The size of each node corresponds to the number of genes in each of these pathways, thickness of connections between nodes corresponds to the number shared genes between pathways and the colors of the nodes shows the p-value for the given metabolic process.

The growth morphology of bioproduction cell lines is of great importance for culture maintenance and efficiency of industrial bioprocessing. In order to look into gene expression variations correlating with adherent and suspension HEK 293 cell lines, differential expression analysis between adherent and suspension HEK293 progeny cell lines was performed. As results from the overall comparison of transcriptomic profiles of the HEK293 cell lines showed that the parental HEK293 cell line is highly differentiated from all of the progeny cell lines and moreover, that the Freestyle 293-F cell line is very similar to the 293-F cell line, HEK293 and Freestyle 293-F were excluded from this analysis, so as not to skew the data. Enrichment analysis of the differentially expressed genes between adherent (293T and 293E) and suspension (293-H and 293-F) progeny cell lines showed significant expression differences of similar gene sets as in the comparison between progeny cell lines and the parental HEK293 (Figs. 3a, 4a, Supplementary Table S6). For instance, the suspension progeny cell lines had a significant up-regulation of gene sets involved in cellular compartment organization such as cell morphogenesis, cell junction-, cell membrane- and cytoskeleton organization. Interestingly, there is no significant change in the expression of the extracellular matrix organization gene set between suspension and adherent HEK293 progeny cell lines. Perhaps as expected, there was significant differential expression observed for the cell adhesion, cell differentiation, cell morphogenesis and cell motility gene sets. All of the above-mentioned gene sets, including cell adhesion, were up-regulated in suspension HEK293 cells as compared to adherent. When looking at the most significantly differentially expressed genes (adjusted p-value < 0.01) amongst the cell adhesion gene set, many genes of the cadherin superfamily of cell adhesion molecules were found up-regulated in suspension cell lines compared to adherent HEK293 progeny cells (Supplementary Table S7).

In order to evaluate what metabolic impact the differentially expressed genes may have on cells in suspension compared to the adherent state, a generic human metabolic model, HMR2^35^, was used to generate a set of metabolic genes and their assigned pathways to find metabolic pathways with altered expression between adherent and suspension HEK293 progeny cell lines. As shown in Fig. 4b, pathways related to aromatic amino acids and oxidative phosphorylation were significantly down-regulated in suspension cells compared to adherent and had amongst the highest number of differentially expressed genes. Pathways related to retinol, linoleate and nucleotide metabolism were also significantly down-regulated in suspension cell-lines. On the other hand, biosynthesis and metabolism of cholesterol were found to be most significantly up-regulated amongst metabolic pathways in suspension compared to adherent cells. In addition, pathways related to protein modification and fatty acid metabolism (omega-3/6 fatty acid metabolism, fatty acid desaturation, fatty acid biosynthesis and fatty acid elongation) were up-regulated in suspension compared to adherent HEK293 progeny cell lines. All results from the metabolic gene set analysis are provided in Supplementary Table S8.

**Figure 5:**
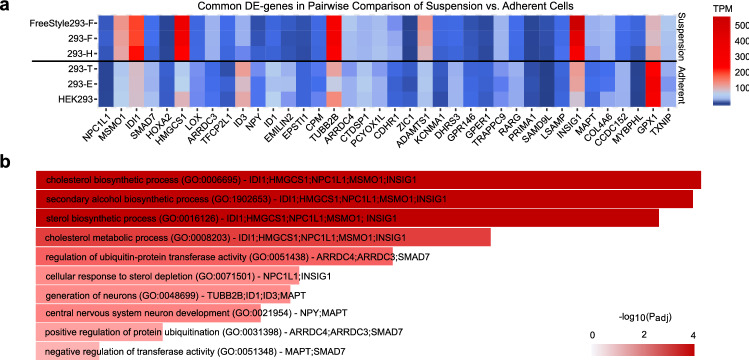
Evaluation of common DE genes between adherent and suspension HEK293 cells identified cholesterol biosynthesis as the main enriched pathway. (**a**) Heat map with TPM values for each DE gene common to all adherent to suspension comparisons. (**b**) The top ten most enriched biological GO terms of the 38 common DE genes between adherent and suspension cells based on gene enrichment analysis. Length and color of bars both show significance of adjusted p-value for the hypergeometric test. Also, genes mentioned in each bar are the genes that belong to enriched GO term and present in the list of 38 DE genes.

Focusing on the pairwise comparisons between HEK293 cell lines, 38 differentially expressed genes were identified common to all adherent to suspension pairwise comparisons (red column in Figs. 3b, 5a, Supplementary Table S5). Three of the genes (ARRDC3, HMGCS1 and PCYOX1L) had the same directional gene copy number variation (gain or loss) compared to gene expression fold-changes (up or down) of all suspension compared to adherent cells, which may at least partly explain the differential expression of these genes between the groups. Gene enrichment analysis of this set of 38 differentially expressed genes between adherent and suspension cells, performed using Enrichr^36^, predicted the cholesterol biosynthetic process pathway as the cellular pathway most affected by this expression variation (Fig. 5b). This result further emphasizes the differential expression between adherent and suspension cells of genes involved in the cholesterol pathway, mentioned above. Among the 38 common differentially expressed genes MSMO1, IDI1, NPC1L1, INSIG1 and HMGCS1 are directly related to cholesterol biosynthesis (GO:0006695 and/or GO:0008203, Fig. 5b). Each of these genes had at least a two-fold increase in expression in the suspension cells compared with the adherent cell-lines. Based on these findings, we sought to predict the effect of the differentially expressed genes between adherent and suspension HEK293 cell lines on the cholesterol biosynthesis of the cells using Ingenuity pathway analysis (IPA). Although the MSMO1, IDI1 and HMGCS1 genes were all up-regulated in 293-F and 293-H compared to HEK293, the down-regulation of the lathosterol oxidase gene (SC5D), which gene product is in downstream steps of the pathway, resulted in a predicted reduction in cholesterol production in 293-F and 293-H cells compared to HEK293 (Supplementary Fig. S7). Comparisons between suspension cell lines (293-F and 293-H) and adherent progeny cell lines (293E and 293T) did not result in any predicted changes in cholesterol biosynthesis (data not shown).

As four out of the 38 differentially expressed genes (LOX, SMAD7, ID1 & TXNIP) have previously been shown to have a role in the epithelial to mesenchymal transition (EMT) pathway^37^, we evaluated the role of this pathway in the transition from adherent to suspension cell growth of these HEK293 cell lines. The normalized expression of a set of EMT markers showed that the parental HEK293 actually had the highest level of expression of various mesenchymal markers (N-cadherin, vimentin and fibronectin) of all the six cell lines (Supplementary Fig. S8). Moreover, when predicting EMT pathway outcomes for suspension cells (293F and 293H) compared to the parental HEK293 strain using IPA, the results predicted reduced EMT in the suspension progeny cells compared to HEK293 (Supplementary Fig. S8). However, suspension cells were predicted to have increased disruption of adherence junctions, which is consistent with the suspension cell phenotype. Taken together the comparison between adherent and suspension HEK293 progeny cell lines suggest that the transition between adherent to suspension cell growth is not equivalent to the epithelial to mesenchymal transition even though several EMT-associated genes may be key to the difference between cell lines. Instead key changes were found associated with cholesterol biosynthesis and fatty acid metabolism.

### Identification of five genes with potential key roles in differences between human adherent and suspension cell lines

**Figure 6:**
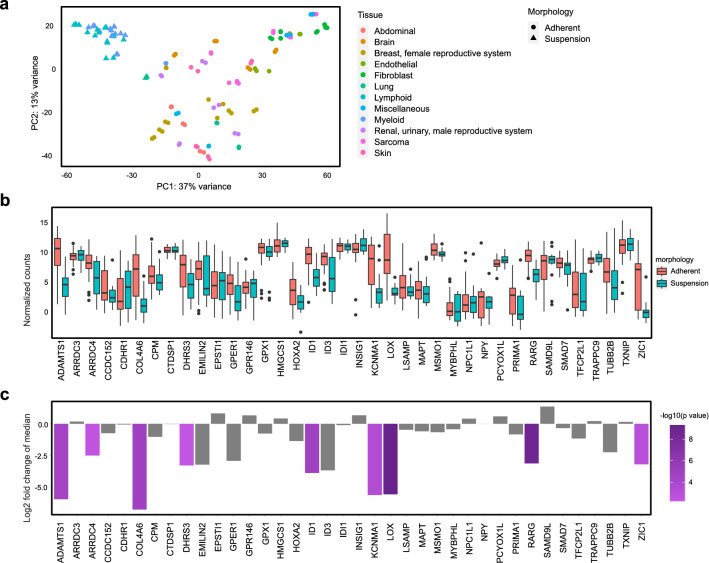
Gene expression validation of the 38 previously identified differentially expressed genes in 63 human cell lines, identified nine significantly differentially expressed genes between suspension and adherent cell lines. (**a**) PCA transcriptomic data of 63 human cell-lines from the Human Protein Atlas shows a clear separation of suspension and adherent cell lines from different tissues. (**b**) Range of normalized counts in HPA cell lines for each of the previously identified 38 genes, differentially expressed between all adherent and suspension HEK293 cell lines. The black line in each box shows median of normalized counts for the gene. (**c**) Genes that are differentially expressed between adherent and suspension cells using a Mann–Whitney U-test, with p-values < 0.01, are highlighted in purple, where length of bars shows logarithmic fold change of median between two groups and the color of bars denotes degree of significance of p-value. Non-significant genes have gray bars.

For identification of key genes involved in the transition from adherent to suspension morphology, expression data from an additional set of 63 different human cell lines deposited in the Human Protein Atlas database^38^ were analyzed. Principal component analysis of these cell lines resulted in clustering of suspension cell lines in a distinct group separated from adherent cell lines (Fig. 6a). However, since most of the suspension cell lines are lymphoid or myeloid-derived this clustering may be a result of the similar origin of the suspension cell lines. Transcription data of the 38 previously identified differentially expressed genes from 47 adherent and 16 suspension cell lines (Supplementary Table S9) was compared between the two groups using a Mann–Whitney U-test^39,40^. Within this set, nine genes (LOX, ID1, ADAMTS1, ZIC1, KCNMA1, DHRS3, RARG, COL4A6 and ARRDC4) had significant different expression levels between adherent and suspension cell lines with p-values < 0.01 (Fig. 6b,c). Four of these genes (ADAMTS1, KCNMA1, COL4A6 and ARRDC4) had the opposite directional change in the extended data set compared to the differential expression between only HEK293 strains. Based on these findings, the remaining five genes (LOX, ID1, ZIC1, DHRS3 and RARG), which showed a consistent down-regulation in suspension cell lines compared to adherent cells, may play important roles in the morphological differences between the adherent and suspension cell lines.

## Discussion

Due to the extensive usage of HEK293 cells as a bioproduction platform for pharmaceutical proteins and AAV vectors, characterization of the HEK293 genome and transcriptome is relevant for bioprocess development. A deeper knowledge of the HEK293 genomic and transcriptomic traits can for instance pave the way for more rational cell line engineering approaches, aiming to improve bioproduction efficiency and quality of protein products. As different HEK293 lineages are propagated under different conditions and the observation that immortalized continuously cultured cell lines, such as HEK293, have a high degree of genomic instability with frequent chromosome rearrangements^22,25^ it can be expected that different HEK293 lineages are differentiated at the genomic and transcriptomic level compared to the parental cell line. Here, the genomes and transcriptomes of the original HEK293 along with five progeny cell lineages were analyzed (Fig. 1a). The overall comparison of genomic and transcriptomic profiles confirmed the picture of clonally diverged progeny cells as compared to the parental HEK293 (Fig. 1b,c). As expected, there was a high degree of genomic and transcriptomic similarities of the Freestyle 293-F and 293-F cell lines (Fig. 1b–d). The results presented here indeed show that they are highly similar both on a genomic and transcriptomic level and confirm the previously reported findings that standard propagation of HEK293 cell lines does not alter the genomic profile to a large extent^22^. Furthermore, based on the hierarchical clustering, the adherent progeny cell lines showed a higher degree of divergence from each other compared to suspension cells. This may be a result of the independent transformation and isolation of the 293T and 293E lineages by the stable integration of different viral genes in different labs. The relatively low expression level of EBNA1 observed in 293E cells may be an effect of cultivating cells in the absence of geneticin in this study, in order to minimize differences in cultivation conditions between cell lines. Interestingly, a truncated version of the Large T antigen was also found to be expressed in 293E cells, which has to our knowledge not been reported previously (Fig. 1e and Supplementary Fig. S2). This sequence was likely derived from the pRSVneo plasmid that was co-transfected with pCMV-EBNA1 during the isolation of the 293E c18 clone^31^.

The overall comparison of the genomic and transcriptomics profiles of HEK293 cell lines suggests that the parental HEK293 strain has the highest divergence amongst the cell lines. Common changes in gene copy number gain or loss (Fig. 2 and Supplementary Fig. S3) and consensus differential expression alterations (Fig. 3) were observed amongst all progeny cell lines when compared to HEK293. For instance, a common dense pattern of copy number gain and loss for progeny cell lines was observed on chromosome 13 and 18 (Fig. 2a). Such patterns, found across all or several lineages of HEK293 isolated independently by different methods, suggests a selective advantage for altered copy numbers of such loci in regard to the phenotypes of the cell lines. On chromosome 13, several genes associated with the cytoskeleton (BORA, MZT1, PIBF1, DACH1 and KLHL1) had a copy number gain in all progeny cell lines (Supplementary Table S3). Indeed, consensus gene expression changes associated with cytoskeleton organization were observed between all progeny HEK293 cell lines and HEK293 (Fig. 3a). In addition, BORA, MZT1 and DACH1 were found amongst the 329 genes commonly differentially expressed between all adherent and suspension cell lines (Supplementary Table S3). Moreover, amongst the five genes with high impact SNVs found in all progeny cell lines compared to HEK293, one gene (SGCD) encodes the cytoskeletal protein delta-sarcoglycan. Within the gained region of chromosome 18 common to all progeny cell lines except 293E, there are several cell adhesion molecules (DSC1, DSC2, DSC3, DSG1, DSG2, DSG3 and DSG4), which may render this region prone to gene copy number variation during cell line development. The observed enrichment of cell adhesion GO-terms (GO:0,007,156 and GO:0,098,742) amongst genes with common high/moderate impact SNPs unique to progeny cell lines compared to HEK293 cells also supported common genomic alterations in progeny cell lines involved in cell adhesion. Combined with the observed down-regulation of the entire cell adhesion gene set in progeny cell lines compared to HEK293 (Fig. 3a), results highlight changes in cytoskeleton and cell adhesion during continuous cultivation and cell line development of HEK293 cells. Such traits associated with cell adhesion, cell motility and extracellular matrix organization may result from selective pressure, through inefficiency of detaching the most adherent cells, during single cell cloning. Indeed, the adherent progeny cell lines 293E and 293T are easier to detach by trypsinization compared to the parental HEK293, potentially through altered expression of cell adhesion, cytoskeletal and cell membrane proteins in accordance with the observed consensus changes between progeny cell lines and the parental HEK293.

Moreover, specific genomic regions of more local gain or loss of specific genes were observed, including a loss of fumarate hydratase (FH) gene copies. The loss of FH copies was previously observed for HEK293 by Lin and coworkers and was suggested to play an important part in the transformed phenotype of the cell line^22^. In line with this, our results showed that several of the HEK293 progeny cell lines (293E, 293-F and Freestyle 293-F) were found to only maintain half the number of FH gene copies compared to the original HEK293 (Supplementary Fig. S4), supporting an advantageous loss of the FH gene in the HEK293 cell lineages. Furthermore, a conserved pattern of substantial gain (> 1.5 log2-fold change) of the TLE4 gene and surrounding loci, was observed for all progeny cells except 293E (Supplementary Fig. S4). Interestingly, TLE4 has previously been reported to have both a tumor suppressor function and to be associated with promoting tumor growth in different studies of different cancers^41,42^. Moreover, a significant loss of the ADAM3A pseudogene (< -1 log2-fold change), which has previously been associated with different cancers^43,44^, was observed in all progeny cell lines except 293-H compared to HEK293 (Supplementary Fig. S4). The specific gain of TLE4 and/or loss of ADAM3A loci and their association with tumor development, suggest important functions of these genes in the evolution of HEK293 cell lines, potentially through effects on proliferation and/or evasion of normal cell senescence of progeny cell lines.

In bioproduction processes for pharmaceutical proteins, suspension cell lines enable large-scale cultivation in bioreactors, which is required in order to meet the demands for marketed drugs. However, the adaptation of cells from adherent to suspension growth and the differential cultivation procedures between adherent and suspension cells induces phenotypic changes to the cell lines. In order to develop a deeper understanding of such changes, we evaluated differences in gene expression levels between adherent and suspension progeny HEK293 cells. Consensus differential expression results were found related to up-regulation of genes associated with cell component organization such as membrane, cytoskeleton and cell junction in suspension compared to adherent cells (Fig. 5a). Noteworthy, cell adhesion was found up-regulated in suspension compared to adherent cells. Amongst the most significant differentially expressed genes (adjusted p-value < 0.01) in the cell adhesion gene set (Supplementary Table S7), several members of the cadherin superfamily, including many different protocadherins (PCDH), desmoglein 2 (DSG2) and desmocollin 2 (DSC2) were found significantly up-regulated in suspension cells compared to adherent cells. This family of genes are involved in the formation of adherence junctions between cells^45^. Notably, DSG2 and DSC2 are located in the region on chromosome 18 that had gained genomic copies in all progeny cell lines except 293E compared to the parental strain (Fig. 2a). Moreover, four protocadherin members showed the highest fold-change of up-regulated genes in suspension cells (Supplementary Table S7). The higher expression of such cell adhesion molecules in suspension cell lines compared to adherent progeny HEK293 cells may be explained by the loss of culture dish support to grow on in case of suspension cells. Upon disruption of adhesion interactions with other cells and extracellular matrix, a natural cellular response may be to increase or maintain the expression of adhesion molecules in an attempt to restore such connections. The ability of the suspension cell lines to form cell aggregates during suspension cultivation and the ease of the cells to attach to culture dish surfaces upon cultivation without shaking, can be speculated to support these findings. Such cell adhesion molecules found up-regulated in suspension cell lines may thus be appropriate cell line engineering targets for improved bioprocess performance of suspension cell lines.

Further evaluation of the differentially expressed genes between adherent and suspension HEK293 progeny cell lines, based on metabolic gene set analysis, highlighted changes in biosynthesis of aromatic amino acids and pathways related to lipids and/or cholesterol metabolic processes (Fig. 4b). These metabolic changes could be a result of different growth media compositions used for the cultivation of either adherent or suspension cells that may imply different concentrations of for instance amino acids, glucose or serum. When reducing the number of differentially expressed genes to those that consistently showed differential expression between adherent and suspension cells in pairwise comparisons of all cell lines, the cholesterol and sterol biosynthesis and metabolism pathway were found to be most significantly different between the cell types (Fig. 5b). Moreover, five of the consistently up-regulated genes in suspension HEK293 compared to adherent encode enzymes that have either direct roles in the cholesterol biosynthesis pathway (MSMO1, HMGCS1 and IDI1)^46^, or proteins that are associated with cholesterol metabolism by various processes (NPC1L1 and INSIG1)^47,48^. As suspension cell lines are cultivated under serum free conditions, the increased expression of genes associated with for instance cholesterol in suspension cell lines may be a result of a lower cholesterol content in the medium. However, as cholesterol is a major component of the cell membrane and has an important function for membrane structure and cell signaling^49^, the differential expression of genes associated with cholesterol synthesis and metabolism may also be of importance for the different morphologies between adherent and suspension HEK293 cells. Indeed, previous studies have shown that cholesterol plays a critical role in regulating the formation of cell-to-cell interactions in endothelial cells^50^ and that depletion of cholesterol reduces cell adhesion and increases endothelial cell stiffness^51,52^. Increased cell surface stiffness has been reported for HEK293 cells in suspension compared to adherent state as a result of up-regulation and re-organization of the actin cytoskeleton^53^. This may partly be a result of altered cholesterol levels in the cell membrane since cholesterol is a regulator of the actin cytoskeleton and cholesterol depletion has been shown to induce actin polymerization^54^. Interestingly, the Insulin-induced gene 1 protein (INSIG1), which was up-regulated in suspension compared to adherent HEK293, is a negative regulator of cholesterol synthesis and important for cholesterol homeostasis^48^ and knockout of INSIG1 has previously been shown to result in cholesterol accumulation^55^. Notably, a lower cholesterol biosynthesis in suspension cell lines compared to the original HEK293 strain was indeed predicted using IPA (Supplementary Fig. S7). It should however be noted that this prediction does not take into consideration the effect of INSIG1, instead the predicted reduction in cholesterol biosynthesis in suspension cells compared to the HEK293 cell line is a result of down-regulation of SC5D (lathosterol oxidase). From a bioprocess perspective, differences in intracellular cholesterol synthesis and metabolism may also be of interest with regards to the secretory capacity of a cell line since previous findings has shown that cholesterol is essential for ER to Golgi transport within the secretory pathway^56^ and that secreted productivity of CHO cells increases upon elevated intracellular cholesterol levels, through silencing of INSIG1, possibly due to increasing the volume of the Golgi compartment^57^. It would therefore be of interest to gain further knowledge about the cholesterol content and distribution within HEK293 cell lines and potentially evaluate if this pathway can be targeted for enhanced bioproductivity without having a deleterious impact on suspension growth or cell morphology.

Four of the 38 genes (ID1, SMAD7, TXNIP and LOX) that were consistently differentially expressed between adherent and suspension HEK293 have previously been annotated to play a role in epithelial to mesenchymal transition (EMT)^37^, the event where stationary epithelial cells lose their cell–cell adhesion and change into motile and invasive mesenchymal cells^58^. However, when evaluating the expression of common markers for mesenchymal and endothelial phenotypes as well as predicting the outcome of the EMT pathway using IPA, the parental HEK293 strain showed the most mesenchymal-like phenotype whereas suspension cell lines were predicted to have reduced transition from epithelial to mesenchymal phenotype compared with HEK293 (Supplementary Fig. S8). These results indicate that the suspension adaptation of HEK293 lineages does not follow the EMT pathway.

Altogether nine of the 38 identified genes (LOX, ID1, ADAMTS1, ZIC1, KCNMA1, DHRS3, RARG, COL4A6 and ARRDC4), with differential expression between all adherent and suspension comparisons of HEK293 (Fig. 5a), were shown to have significantly different expression between adherent and suspension cells also in an extended validation of the genes in a set of 63 human cell lines from the HPA database^38^ (Fig. 6c). Five of these genes (LOX, ID1, ZIC1, DHRS3 and RARG) showed a consistent expression profile (same direction of up- or downregulation) between adherent and suspension cells compared to the results presented in Fig. 5b, suggesting a key role of these genes in the morphologies of adherent and suspension human cell lines. In support of this hypothesis, up-regulation of ID1, as found in adherent cells compared to suspension cell lines, has been associated with the mesenchymal-to-epithelial transition^59^. Moreover, ID1 silencing has also been shown to significantly reduce adhesion of neural stem cells^60^ and conversely, increased ID1 expression in epithelial cells has been related to increased adhesion^61^. In addition, lysyl oxidase (LOX), an enzyme responsible for the covalent cross-linking between elastin and collagen in the extracellular matrix, has been shown to be important for cell–matrix adhesion formation, supporting the adherent phenotype of adherent cells but is also associated with cell invasion and induction of EMT^62,63^. Besides the EMT-related genes, the additional three genes (RARG, ZIC1 and DHRS3), consistently up-regulated in adherent cells compared to suspension cell lines, have previously been associated with increased cell adhesion through the retinoic acid signaling pathway^64–67^. In line with this, retinol metabolism was found to be down-regulated in suspension cells in the metabolic gene set analysis (Fig. 4b).

## Conclusions

Our study has outlined the genomic and transcriptomic variations between six industrially relevant HEK293 cell lines, in an attempt to improve the understanding of their respective differences in phenotype. We report a selective pressure to develop certain expression profiles during the evolution and continuous cultivation, evidenced by the numerous genes and pathways detailed here. The key common changes between HEK293 and its progeny cell lines involve in particular cell membrane proteins and processes related to cell adhesion, motility and the organization of various cellular components such as the cytoskeleton and extracellular matrix. In addition, changes associated with differences between adherent and suspension cell growth in particularly involve changes in cell adhesion protein expression, cholesterol metabolism and a set of six key genes (RARG, ID1, ZIC1, LOX and DHRS3) with potentially key roles in the differentiation between the two groups. These results could be of importance when pursuing further cell line engineering or bioprocess optimization of these and other human cell lines.

## Methods

### Cell cultivation for DNA and RNA preparation

The adherent cell lines HEK293 (ATCC-CRL-1573), HEK293T (ATCC-CRL-3216) and 293E (ATCC-CRL-10852) were obtained from ATCC and propagated in DMEM (D6429) supplemented with 10% FBS at 37 °C in a humidified incubator with 5% CO_2_ in air. Suspension cell lines 293-F, 293-H and Freestyle 293-F (Gibco) were obtained from Thermo Fisher Scientific and cultivated in 293 SFM II medium (Gibco) supplemented with Glutamax at a final concentration of 4 mM (Gibco). Suspension cells were cultivated in 125-ml Erlenmeyer shake flasks (Corning) at 37 °C and 120 rpm in a humidified incubator with 8% CO_2_ in air. All cells were propagated from frozen stocks for no longer than 20 passages.

### RNA and DNA preparation and sequencing

Adherent cells were detached by trypsinization and both adherent and suspension cells were harvested by centrifugation. Genomic DNA was extracted using the Blood and Cell Culture DNA Mini Kit (Qiagen) according to the manufacturer’s guidelines and concentrations were determined by using a NanoDrop ND-1000 spectrophotometer (Thermo Scientific). Genome sequencing was performed at the National Genomics Infrastructure (Scilifelab, Solna, Sweden) using the Illumina HiSeq X platform. For RNA extraction, cells grown in log phase from three biological replicates were collected (derived from successive propagations). Cell pellets were resuspended in RNAlater Stabilization Solution (Invitrogen) according to the manufacturer’s recommendations until RNA extraction. Total RNA was extracted from three replicates of each cell line using Qiagen’s RNeasy plus Mini Kit according to the manufacturer’s instructions. Concentrations were determined with a NanoDrop ND-1000 spectrophotometer and RNA quality was assessed on a 2100 Bioanalyzer (Agilent Technologies) using RNA 6000 Nano chips (Agilent Technologies). All samples had an RNA integrity number of at least 9.9. RNA sequencing was performed at GATC (Konstanz, Germany) using the Inview Transcriptome Advance service and an Illumina HiSeq instrument.

### DNA-sequencing analysis

Genome sequencing reads were aligned to the reference (human_g1k_v37.fasta) using bwa (0.7.12)^68^. The raw alignments were then deduplicated, recalibrated and cleaned using GATK (version 3.3–0-geee94ec, gatk-bundle/2.8)^69^. Quality control information was gathered using Qualimap (v2.2)^70^. SNVs and indels have been called using the GATK HaplotypeCaller^69,71^. These variants were then functionally annotated using snpEff (4.1) and snpEff reference GRCh37.75^72^. The Piper pipeline from the National Genomics Infrastructure was used^73^. The correlation between BAM files was assessed using multibamsummary and its plotCorrelation function from deepTools2^74^. Spearman was used to calculate correlation coefficients between samples, and the clusters are joined with the nearest neighbor. The R package seqCAT^75^ was used to compare SNVs between samples, its compare_profiles function mode parameter was set to the default value “intersection”. The heatmap in Supplementary Fig. S1 was based on the similarity scores between the cell lines and Euclidean distances^76^. To compare the Large T antigen sequences of 293T and 293E, unmapped reads were extracted to new bam files using SAMtools^77^, converted to fastq with BEDTools^78^, and de novo assembled with MEGAHIT^79^. NCBI BLAST was used to identify the Large T antigen in the assembled contigs. To evaluate and visualize copy number variations, CNVkit^80^ was used with its whole-genome sequencing method, cbs segmentation^81,82^ and the HEK293 alignment as reference. GO enrichment analysis of genes with high or moderate impact SNPs was performed using PANTHER classification system^83^.

### RNA-sequencing data

Kallisto^84^ was used to quantify transcripts by pseudo-alignment based on human genome assembly version GrCh37. Log transformed normalized data by DESeq2 was used for cell line clustering and calculation of Euclidean distances of samples. The expression comparison of the viral elements was based on normalized counts from DESeq2. Significant testing of differential mRNA expression of E1A/B elements was done by Welch two sample t-test^85^. For differential expression analysis, raw count data from Kallisto was imported using the tximport package^86^ and analyzed with DESeq2^87^. Wald tests were used to calculate p-values, and the BH method was used for multiple testing correction. Throughout the article a gene is considered differentially expressed if log2- fold change >  ± 1 and FDR < 0.05. In the differential expression analysis between adherent and suspension cells, all suspension cell-lines were compared to all adherent cell-lines, and additionally, all pairwise combinations between suspension and adherent cell-lines were evaluated. For evaluating differential expression of 38 common differentially expressed genes between adherent and suspension HEK293 cell lines in a set of 63 human cell lines, RNA-seq data from each cell line deposited in the HPA database was used. Based on the growth characteristics, cells were divided into two groups of adherent and suspension cells. A Mann–Whitney U-test was used to compare normalized counts based on library size between the two groups for each of the 38 differentially expressed genes^39,40^.

### Gene set analysis

To discover significant alterations of gene sets and metabolic pathways between HEK293 cell lines, the Piano package in R was used^88^. The adjusted p-values and fold changes from the differential expression was used in combination with a gene set collection based on “goslim_generic Biological Process”. The heatmap for the progeny cells lines vs. HEK293 was based on the consensus score calculated based on GO term rank aggregation in Piano for each directionality from all pairwise gene set statistics calculations with Wilcoxon rank-sum test. The heatmap for suspension cells (293-H, 293-F) vs. adherent cells (293E, 293T) was based on the consensus score from gene set statistics calculations with mean, median, sum, Stouffer and tailStrength tests and was calculated with Piano’s consensusHeatmap function. To produce the network plot, gene sets were exported from HMR2^89^. For finding differentially expressed pathways of genes between adherent and suspension cell lines, we used the Wilcoxon statistical test and filtered gene sets with adjusted p-value lower than 0.05 as significantly changed. In addition, for gene set analysis of 38 common DE genes between adherent and suspension cell lines we used EnrichR and GO biological process as gene set collection^36^.

### Ingenuity pathway analysis

In order to predict the pathway changes between cell lines based on differentially expressed genes from pairwise comparisons, ingenuity pathway analysis (IPA, QIAGEN Inc.,) was performed. To consider a gene as differentially expressed we used log2 fold change > 1 or < 1 and adjusted p-value < 0.05. For filtering results of gene set analysis by IPA we used Benjamini–Hochberg multiple testing corrected p-values lower than 0.05 to find gene sets with a different expression pattern.

## Supplementary information

Supplementary Tables.

Supplementary Figures.

## Data Availability

Genomics and transcriptomics data is available at Sequence Read Archive (SRA)—BioProject: PRJNA565658.
