# Author Correction: Evolution from adherent to suspension: systems biology of HEK293 cell line development

**DOI:** 10.1038/s41598-021-85105-9

**Published:** 2021-03-02

**Authors:** Magdalena Malm, Rasool Saghaleyni, Magnus Lundqvist, Marco Giudici, Veronique Chotteau, Ray Field, Paul G. Varley, Diane Hatton, Luigi Grassi, Thomas Svensson, Jens Nielsen, Johan Rockberg

**Affiliations:** 1 KTH ‑ School of Engineering Sciences in Chemistry, Biotechnology, and Health, Dept. of Protein Science, Royal Institute of Technology, 106 91 Stockholm, Sweden; 2 Department of Biology and Biological Engineering, Chalmers University of Technology, 412 96 Gothenburg, Sweden; 3 Biopharmaceutical Development, BioPharmaceuticals R&D, AstraZeneca, Milstein Building, Granta Park, Cambridge, CB21 6GH UK; 4 GammaDelta Therapeutics Ltd, White City Place, London, W12 7FQ UK; 5 Kymab, Babraham Research Campus, Cambridge, CB22 3AT UK; 6 NBIS ‑ Bioinformatics Systems Biology Support, Chalmers University of Technology, 412 96 Gothenburg, Sweden; 7 Novo Nordisk Foundation Center for Biosustainability, Technical University of Denmark, 2800 Kongens Lyngby, Denmark

Correction to: *Scientific Reports* 10.1038/s41598-020-76137-8, published online 04 November 2020

This Article contains errors in the Results section, under subheading ‘Genomic and transcriptomic profiling indicate clonal divergence between parental HEK293 and its progeny’, where the Authors refer to the opposite gene name of viral genes ‘E1A’ and ‘E1B’.

“A comparison of mRNA levels of the viral element E1A showed significantly (p < 0.05) higher expression in HEK293 compared to both 293T and Freestyle 293-F. In addition, both 293E and 293-H had significantly higher expression than 293T, 293-F and Freestyle 293-F. Further, 293-F had significantly higher expression than Freestyle 293-F. (Supplementary Fig. S1) The analysis of the viral element E1B showed that 293-F had significantly higher expression (p < 0.05) than 293-H and Freestyle 293-F (Supplementary Fig. S1).”

should read:

“The analysis of the viral element E1A showed that 293-F had significantly higher expression (p < 0.05) than 293-H and Freestyle 293-F (Supplementary Fig. S1). A comparison of mRNA levels of the viral element E1B showed significantly (p < 0.05) higher expression in HEK293 and 293E compared to both 293T and Freestyle 293-F. In addition, 293-H had significantly higher expression than 293T, 293-F and Freestyle 293-F. Further, 293-F had significantly higher expression than Freestyle 293-F. (Supplementary Fig. S1).”

As a result, in Figure 1e, the data bars for ‘E1A’ and ‘E1B’ are reversed. The correct Figure 1e appears below as Figure 1. The Figure legend is correct.

**Figure 1:**
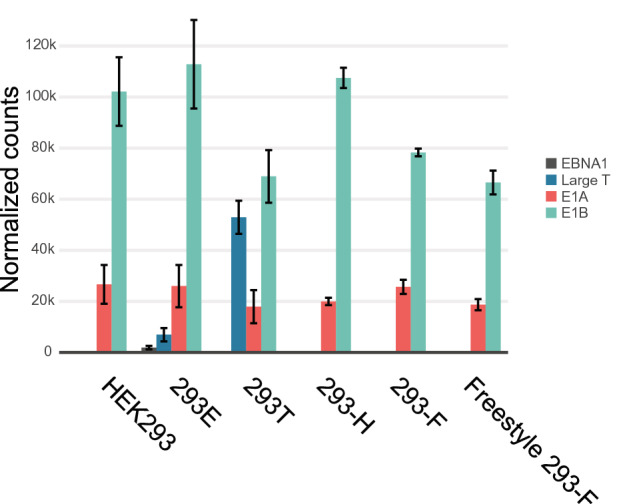
A correct version of the original Figure 1e.

Additionally, in Supplementary Figure S2a, the graphs for ‘E1A’ and ‘E1B’ are reversed. As a result, the Figure legend,

“(**a**) Box plots of E1A and E1B RNA-expression data from the six HEK293 cell lines. A Welch two sample T-test showed significantly (*p < 0.05, **p < 0.01 and ***p < 0.001) higher in E1A expression levels in HEK293, 293E and 293-H cell lines compared to others, whereas E1B was significantly higher in 293-F compared to Freestyle 293-F and 293-H.”

should read:

“(**a**) Box plots of E1A and E1B RNA-expression data from the six HEK293 cell lines. A Welch two sample T-test showed significantly (*p < 0.05, **p < 0.01 and ***p < 0.001) higher E1A expression levels in 293-F compared to Freestyle 293-F and 293-H, whereas E1B was significantly higher in HEK293, 293E and 293-H cell lines compared to others.”

The correct Supplementary Figure S2a appears below as Figure 2.

**Figure 2:**
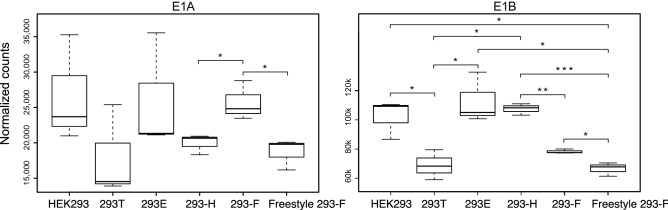
A correct version of the original Supplementary Figure S2a.

