# Supplementary material for: Evolution from adherent to suspension: systems biology of HEK293 cell line development
Source: Sci Rep. 2020 Nov 4;10:18996. doi: 10.1038/s41598-020-76137-8 (PMC7642379; doi:10.1038/s41598-020-76137-8)
Supplement: Supplementary file 2 — Supplementary Figures. [file 41598_2020_76137_MOESM2_ESM.pdf]

Supplemental data

Evolution from adherent to suspension – systems biology of HEK293 cell line development

Magdalena Malm[1*], Rasool Saghaleyni[2*], Magnus Lundqvist[1], Marco Giudici[1], Veronique Chotteau[1], Ray Field[3,4], Paul Varley[3,5], Diane Hatton[3], Luigi Grassi[3], Thomas Svensson[2,6], Jens Nielsen[2,7] and Johan Rockberg[1]

* Joint first authors contributing equally

1 KTH - School of Engineering Sciences in Chemistry, Biotechnology, and Health, Dept. of Protein Science, Royal Institute of Technology, SE-106 91 Stockholm, Sweden
2 Department of Biology and Biological Engineering, Chalmers University of Technology, SE-412 96 Gothenburg, Sweden
3 Biopharmaceutical Development, BioPharmaceuticals R&D, AstraZeneca, Milstein Building, Granta Park, Cambridge CB21 6GH UK
4 GammaDelta Therapeutics ltd, White City Place London W12 7FQ, UK
5 Kymab, Babraham Research Campus, Cambridge CB22 3AT, UK
6 NBIS - Bioinformatics Systems Biology Support, Chalmers University of Technology, SE-412 96 Gothenburg, Sweden
7 Novo Nordisk Foundation Center for Biosustainability, Technical University of Denmark, 2800 Kongens Lyngby, Denmark

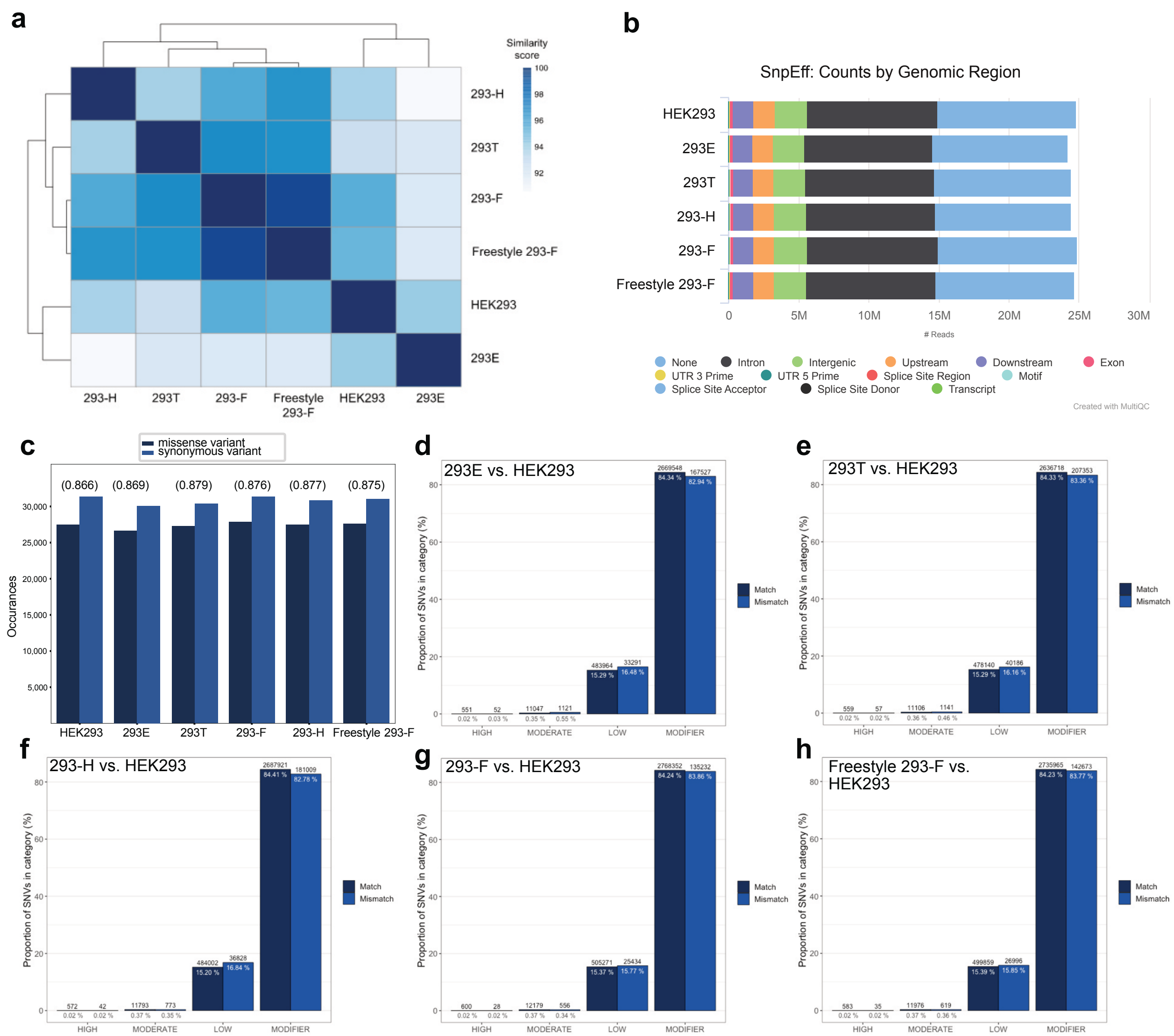

Figure S1: SNV comparisons between the cell lines. (a) Cluster heatmap based on similarity scores calculated using seqCAT. A darker blue color indicates a higher similarity score and thus more similar samples. (b) Counts of variants annotated by SnpEff divided by genomic region. Plot generated by MultiQC[1]. (c) The number of missense and synonomous SNVs in all cell lines compared to the reference genome. On average, the missense/synonymous variant ratio (numbers within brackets) for all cell lines were 0.87 (ranged between 0.866 and 0.879). (d-h) Pairwise comparisons between HEK293 and progeny cell lines. The x-axis shows the four putative impact categories for the SNVs: HIGH is a variant expected to have a disruptive impact on the protein, for example protein truncation or loss of start/stop codon; MODERATE is a non-disruptive variant that might change protein effectiveness, for example a missense variant; LOW is a variant that is not expected to change protein behavior, for example a synonymous mutation; MODIFIER is typically a non-coding variant. Interestingly, the highest number of mismatches in the more impactful high and moderate SNV categories was seen for the two adherent cell lines 293-E and 293-T. Combining these two categories, these two cell

lines had 1173 and 1198 mismatch SNVs compared to HEK293, respectively. The suspension cell lines had the following numbers of mismatches in these categories: 815 (293-H); 584 (293-F); 654 (Freestyle 293-F). The ratios between variants categorized as "missense_variant" and "synonymous_variant" for mismatches between the progeny cell lines and parental cell line were: 0.98 (293E), 1.04 (293T), 1.07 (293-H), 1.01 (293-F), and 1.03 (Freestyle 293-F). On average, the missense_variant/synonymous_variant ratio for all cell lines were 0.87 when compared to the reference genome (ranged between 0.866 and 0.879).

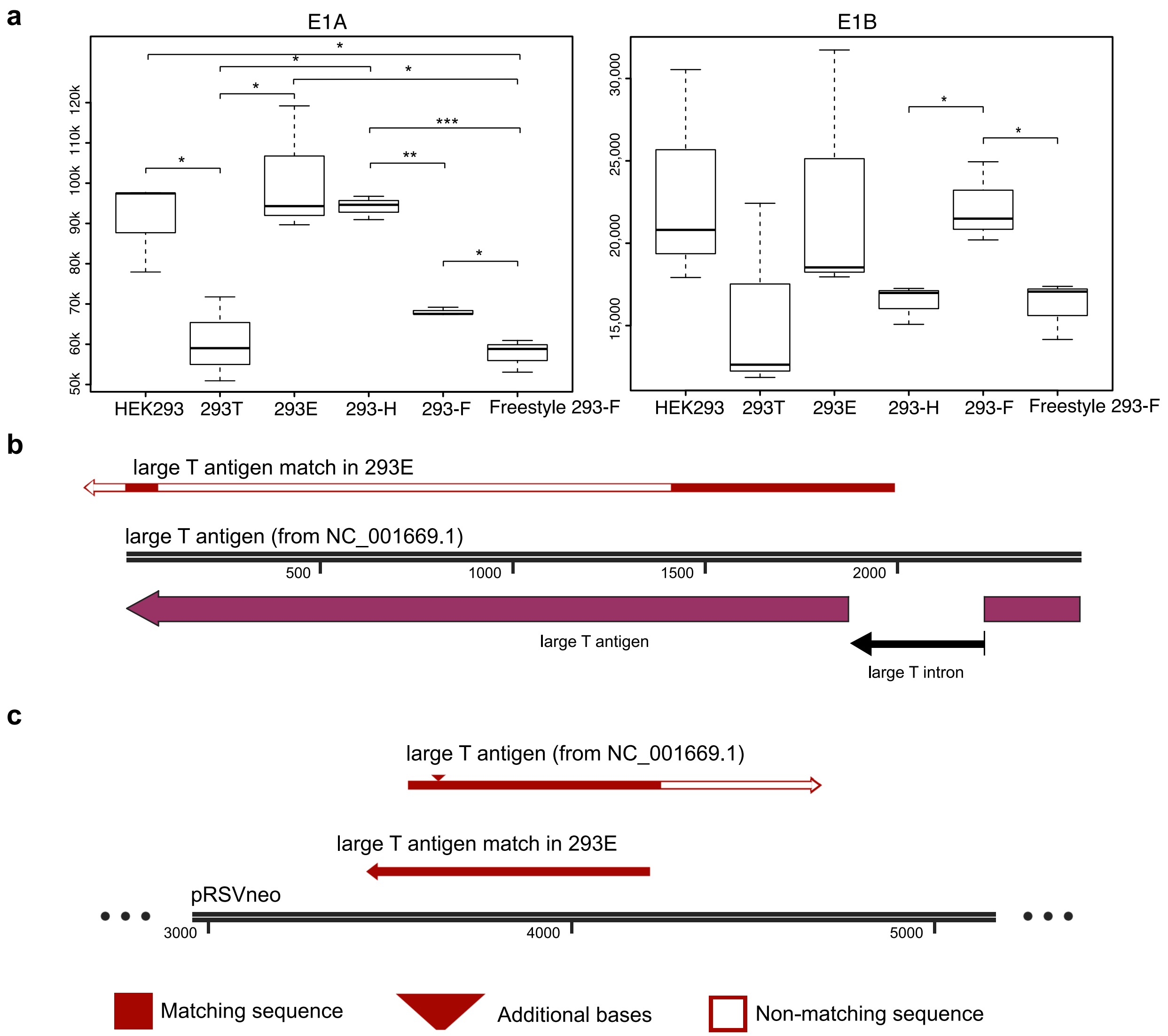

Supplementary Figure S2: Expression and mapping of viral elements of HEK293 cells. Related to Figure 1. **(a)** Box plots of E1A and E1B RNA-expression data from the six HEK293 cell lines. A Welch two sample T-test showed significantly (* $p<0.05$, ** $p<0.01$ and *** $p<0.001$) higher in E1A expression levels in HEK293, 293E and 293-H cell lines compared to others, whereas E1B was significantly higher in 293-F compared to Freestyle 293-F and 293-H. **(b)** Alignment of the assembled genomic contig of 293E with sequence matching LargeT against the SV40 large T sequence (NC_001669.1). **(c)** Alignment of the Large T genomic contig of 293E against the pRSVneo plasmid (Addgene database).

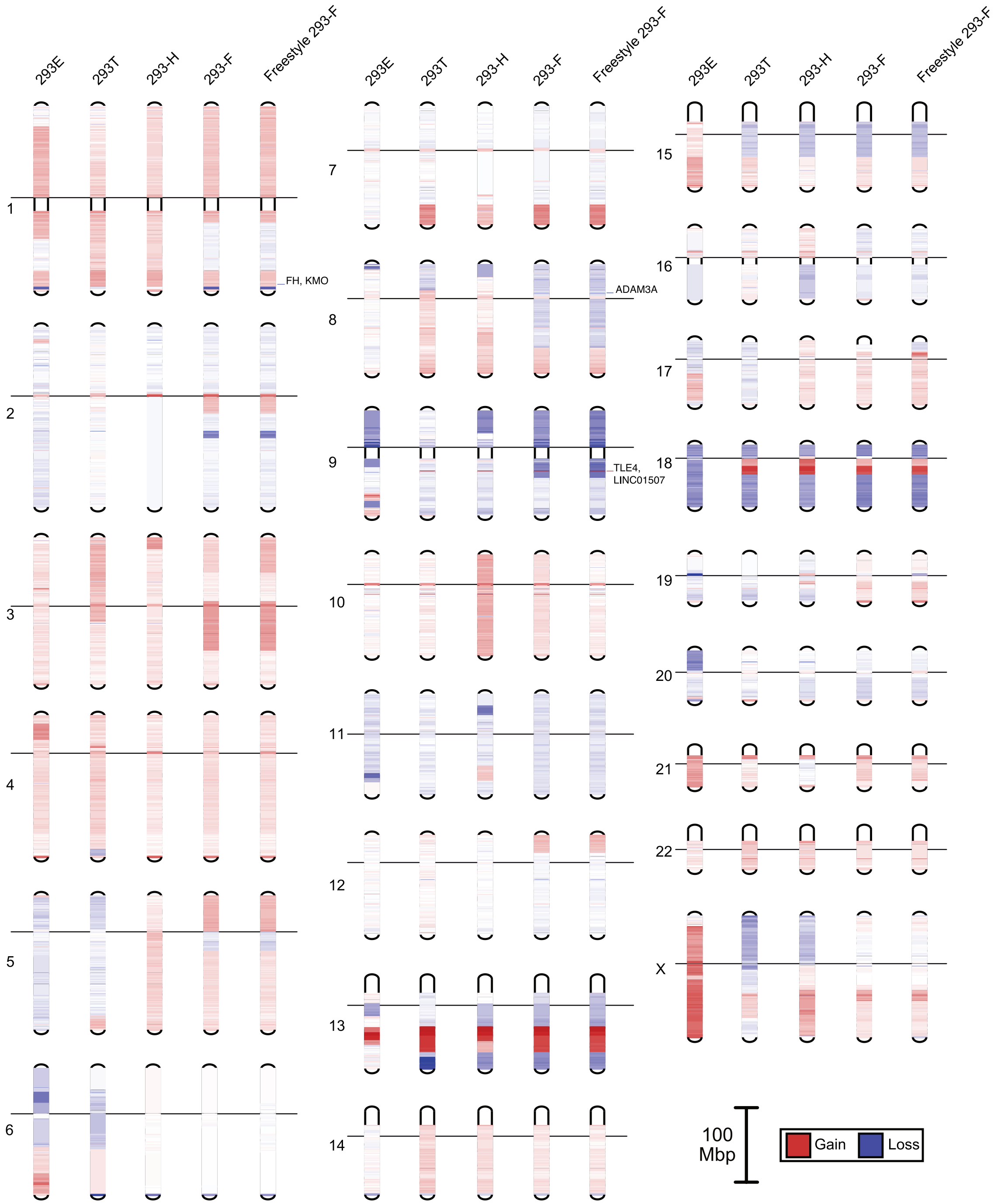

Supplementary Figure S3. Copy number variation analysis of HEK293 progeny cells compared to the parental HEK293 revealed conserved patterns of copy number gain and loss. Related to Figure 2. Genomic copy number gain (red) or loss (blue) of all chromosomes of progeny HEK293 cell lines compared to parental HEK293 cells. The black line indicates the centromere position of each chromosome.

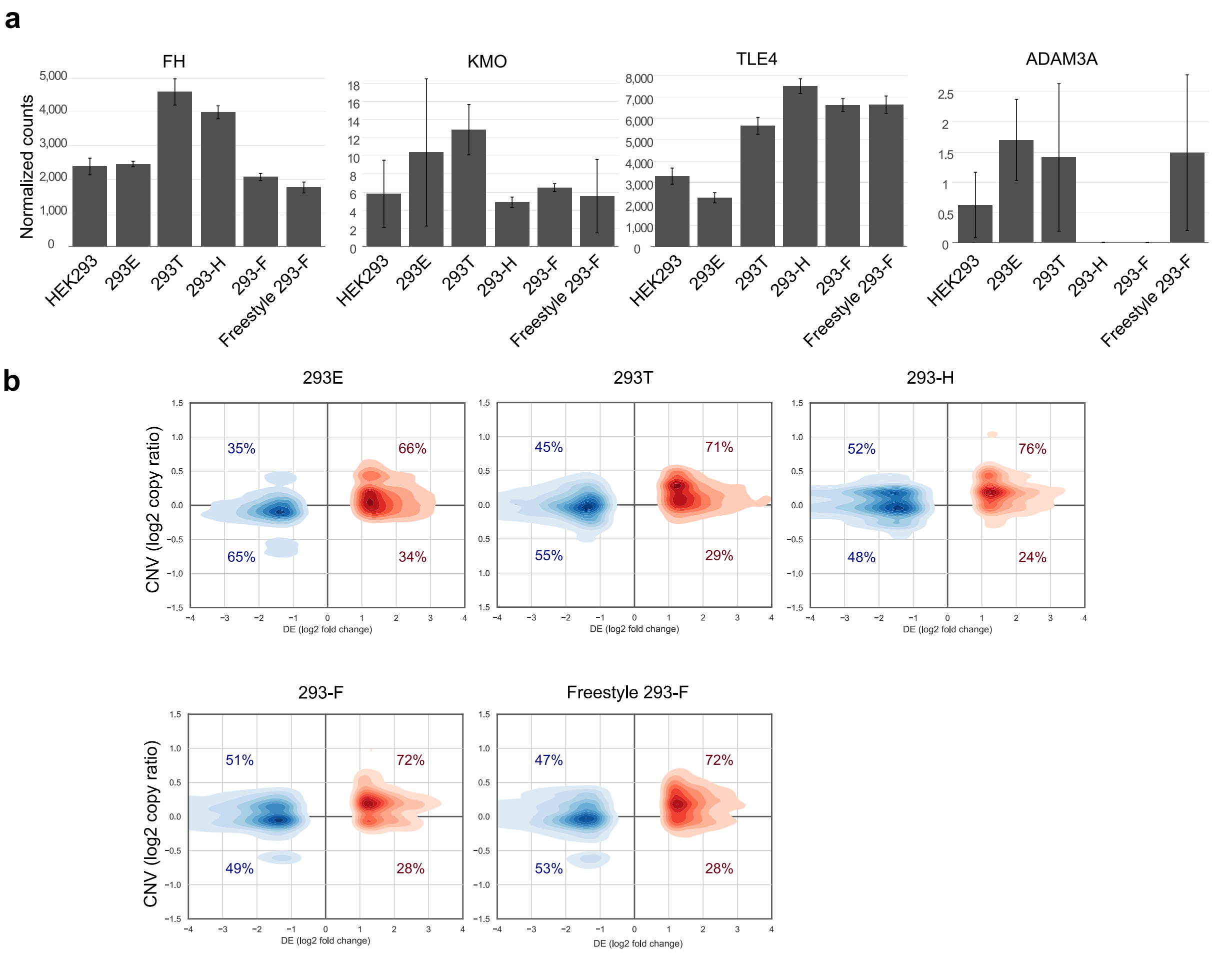

Supplementary Figure S4. Additional copy number variation data. Related to Figure 2 and 3. (**a)** Normalized counts (DESeq2 median of ratios) with standard deviations (n = 3) of FH, KMO, TLE4 and ADAM3A transcripts for each HEK293 cell line based on RNAseq data. **(b)** Density plot of expression fold changes (based on RNAseq data) versus genomic copy number fold-changes of all differentially up-regulated (red) and down-regulated (blue) genes (log2 fold-change >+/-1, adjusted p-value <0.05) for all progeny cell lines compared to the parental HEK293. The percentages correspond to the fraction of up-regulated (red) and down-regulated (blue) differentially expressed genes that were identified with positive respectively, negative gene copy number fold-changes.

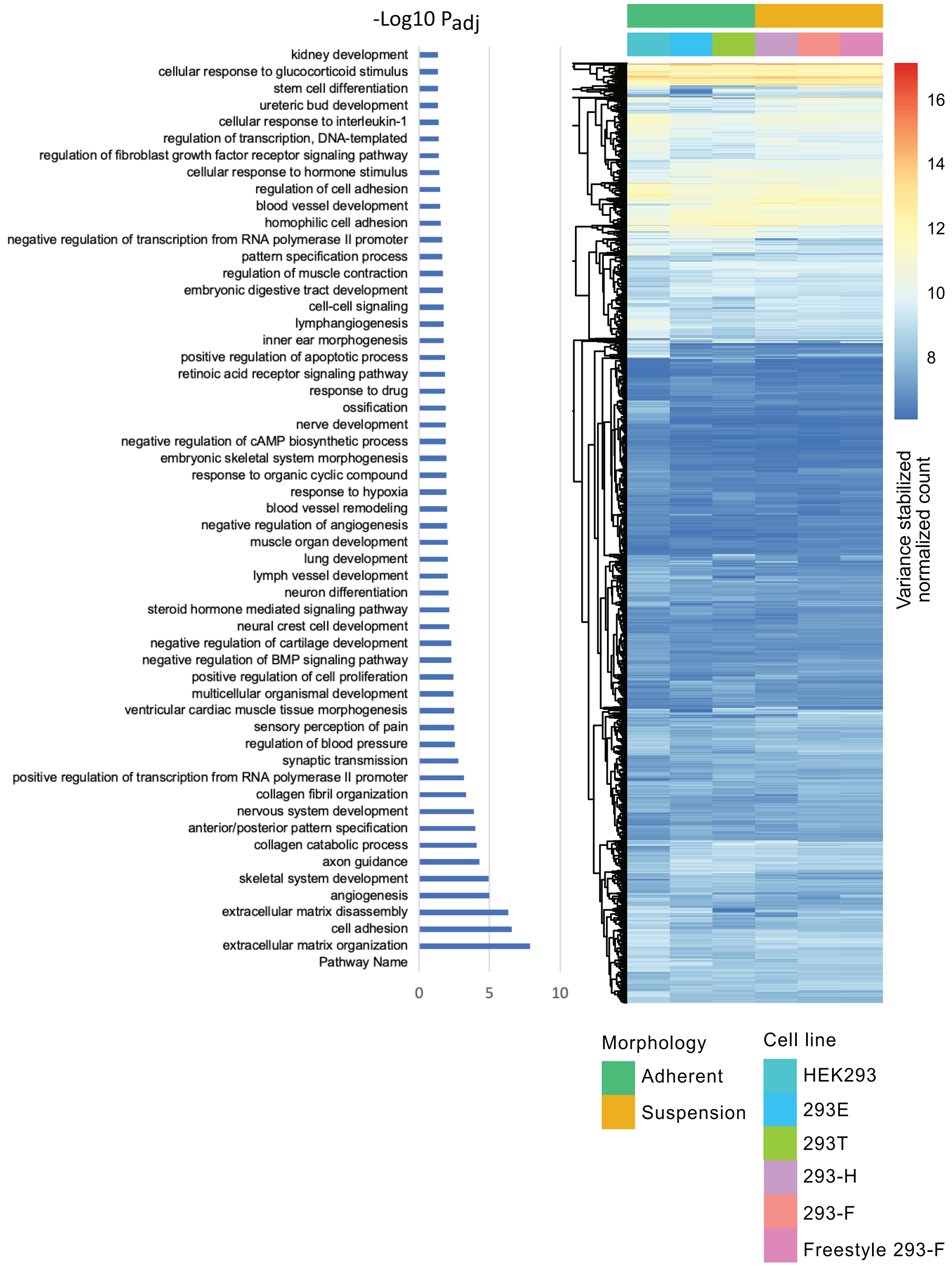

Supplementary Figure S5. Heat map showing normalized counts (variance stabilized normalized counts) of all genes differentially expressed (log2-fold change >+/- 1, Padj< 0.05) in at least one pairwise comparison (in total 4739 genes) between all six HEK293 cell lines. Comparing expression of these genes across all cell-lines highlights more similarity in the expression pattern of progeny cells compared to the parental HEK293 cell line. The most significantly enriched biological GO terms of differentially expressed genes are related to extracellular matrix organization and cell adhesion.

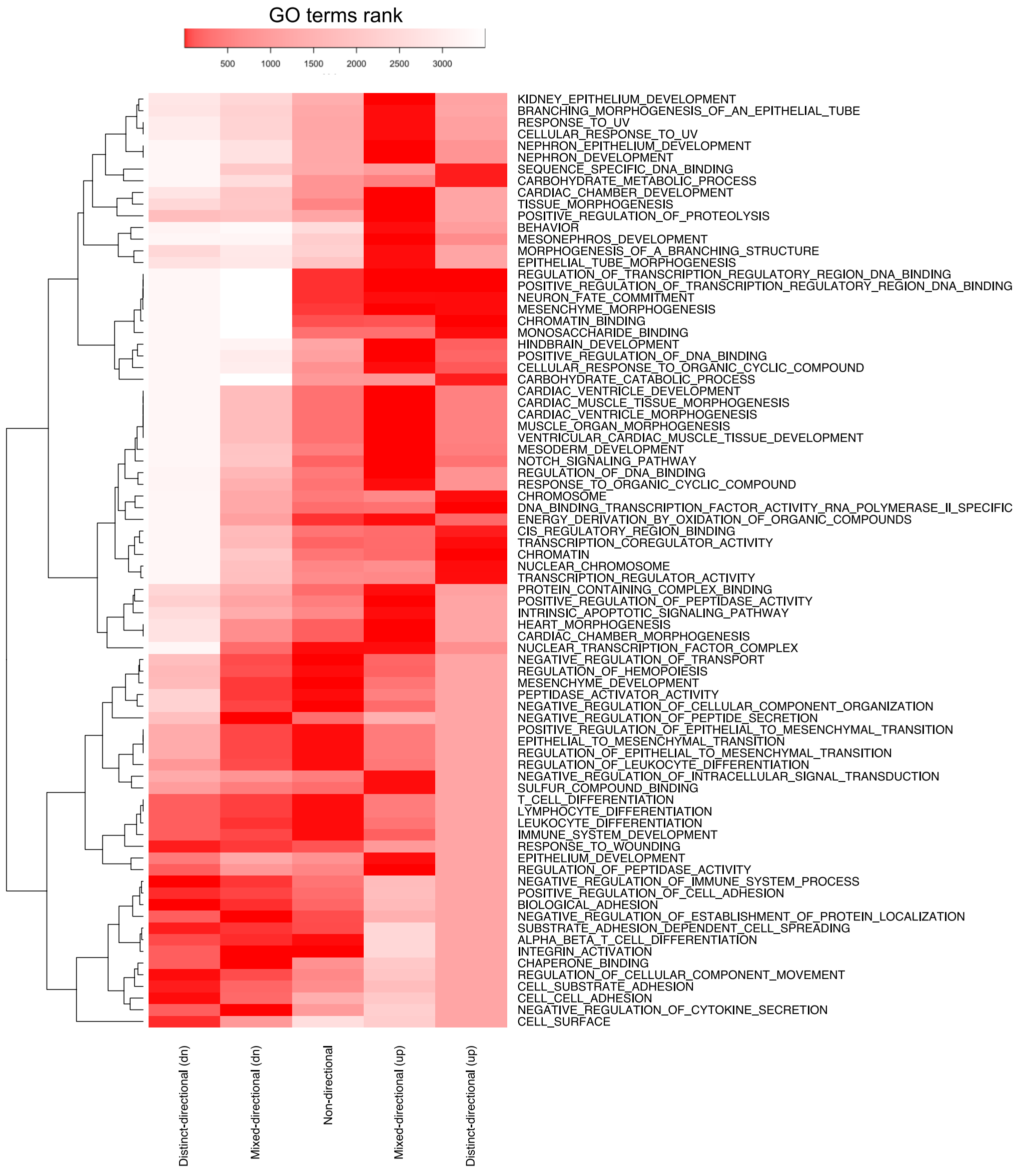

Supplementary Figure S6. Gene set analysis of 329 common differentially expressed genes in pairwise comparison of progeny cells against parental HEK293. Even though no gene set was significantly altered (padj < 0.05) in this limited data set, the heat map emphasizes the involvement of gene sets related to cell surface, cell adhesion and epithelial to mesenchymal transition amongst the 329 genes commonly differentially expressed between progeny and parental HEK293 strains.

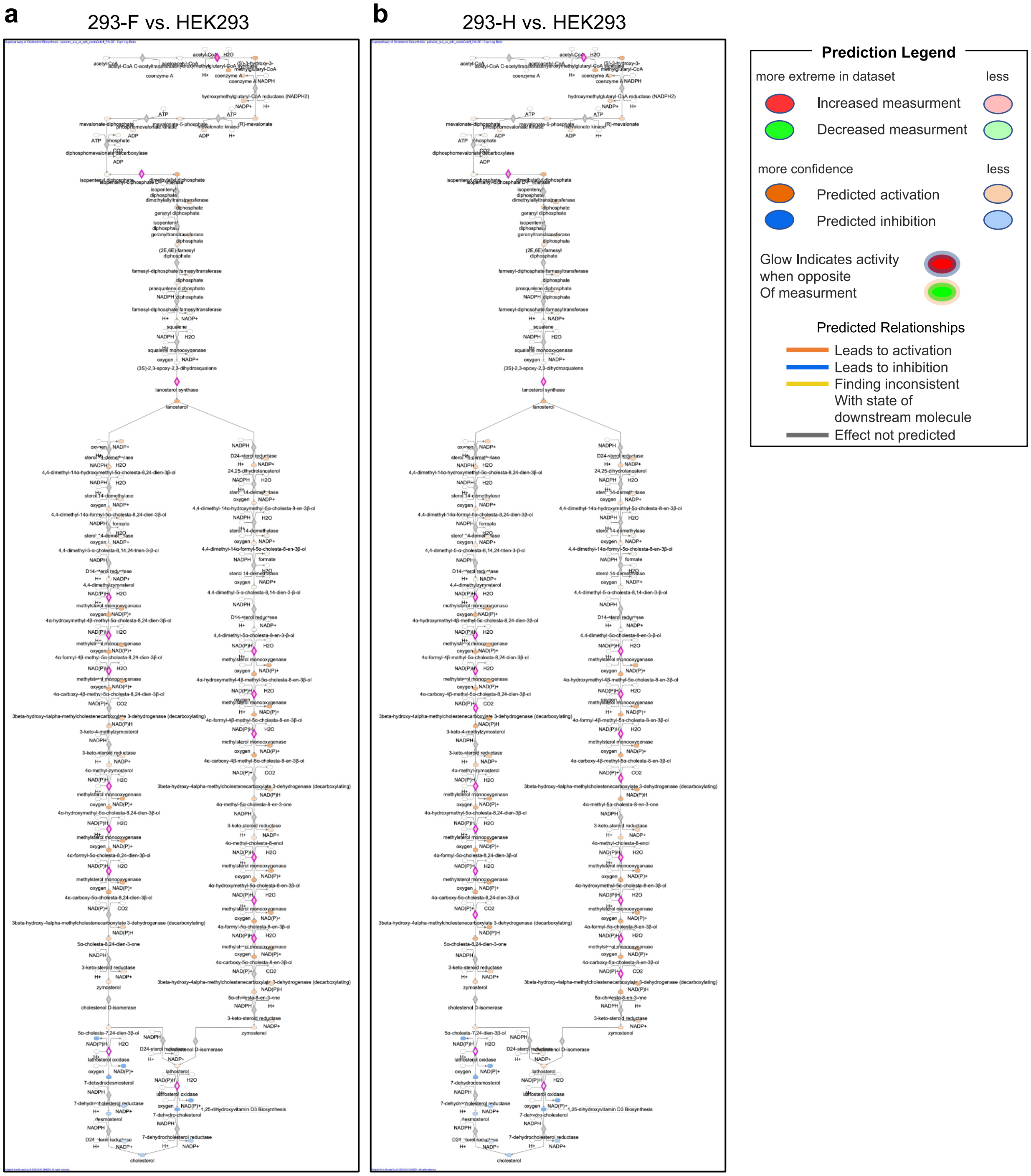

Supplementary Figure S7. Ingenuity Pathway Analysis (IPA) predicted downregulation of cholesterol biosynthesis in 293-F and 293-H cell-lines in comparison with HEK293. The overall cholesterol production in (a) 293-F and (b) 293-H was predicted to be reduced compared to HEK293. Although some biomarker genes for cholesterol production (MSMO1, IDI1 and HMGCS1) were up-regulated in 293-F and 293-H compared to HEK293, the down-regulation of lanosterol oxidase gene (SC5D), which is involved in downstream steps of the pathway, resulted in a predicted decreased cholesterol production in 293-F and 293-H.

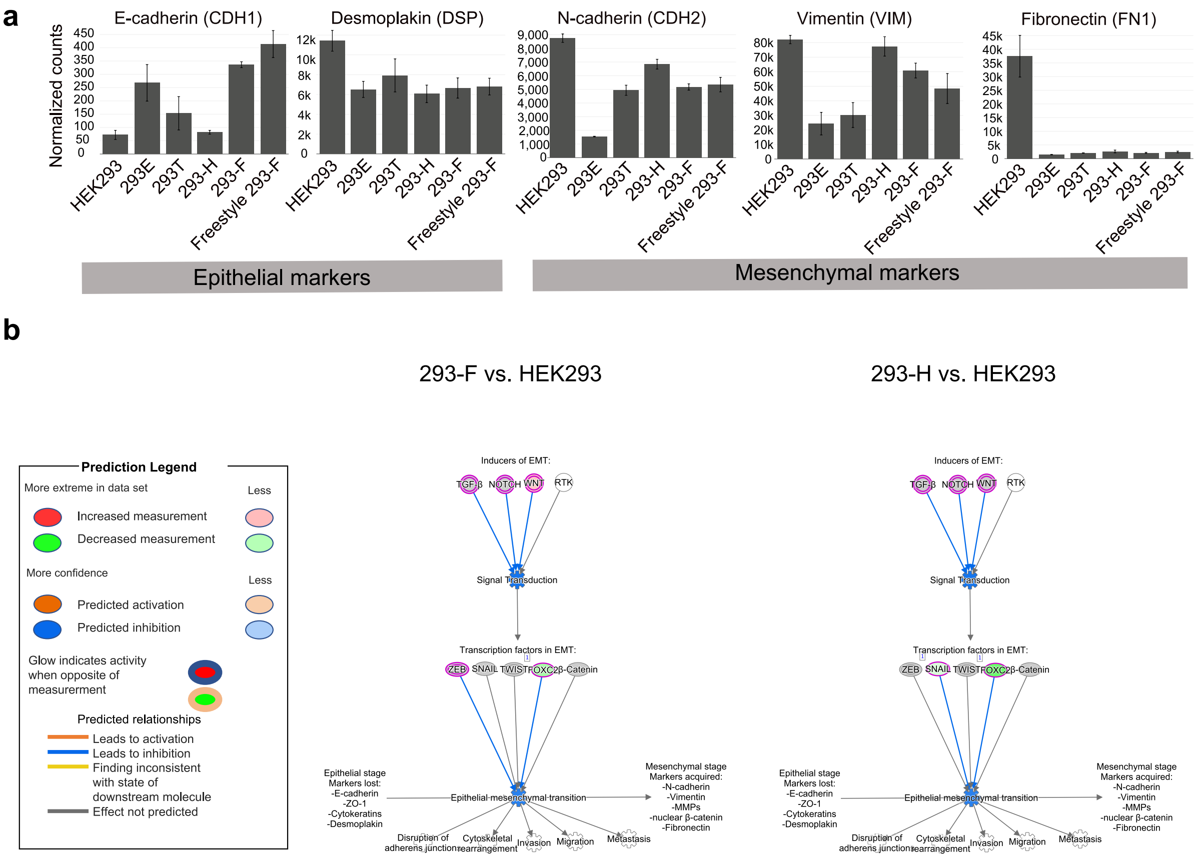

Supplementary Figure S8. Evaluation of EMT-related genes and pathways in HEK293 cell lines suggested reduced EMT activity in progeny suspension cell lines compared to parental HEK293. (a) Normalized counts (DESeq2 median of ratios) and standard deviations (n = 3) of common epithelial markers (E-cadherin and desmoplakin) and mesenchymal markers (N-cadherin, vimentin and fibronectin) in HEK293 cell lines based on RNAseq data. (b) Ingenuity pathway analysis of suspension cell lines 293-F and 293-H compared to the parental HEK293 based on differentially expressed genes.

1. Ewels, P., Magnusson, M., Lundin, S. & Käller, M. MultiQC: summarize analysis results for multiple tools and samples in a single report. *Bioinformatics* **32**, 3047–3048 (2016).
